# Tropical forests as drivers of lake carbon burial

**DOI:** 10.1038/s41467-022-31258-8

**Published:** 2022-07-13

**Authors:** Leonardo Amora-Nogueira, Christian J. Sanders, Alex Enrich-Prast, Luciana Silva Monteiro Sanders, Rodrigo Coutinho Abuchacra, Patricia F. Moreira-Turcq, Renato Campello Cordeiro, Vincent Gauci, Luciane Silva Moreira, Fausto Machado-Silva, Renata Libonati, Thairiny Fonseca, Cristiane Nunes Francisco, Humberto Marotta

**Affiliations:** 1 Ecosystems and Global Change Laboratory (LEMG-UFF), International Laboratory of Global Change (LINCGlobal), Biomass and Water Management Research Center (NAB-UFF), Niterói, Brazil; 2 Physical Geography Laboratory (LAGEF-UFF), Department of Geography, Graduate Program in Geography, Fluminense Federal University (UFF), Niterói, Brazil; 3 Graduate Program in Geosciences (Environmental Geochemistry), Fluminense Federal University, Niterói, Brazil; 4 National Marine Science Centre, Faculty of Science and Engineering, Southern Cross University, PO Box 4321, Coffs Harbour, 2450 NSW Australia; 5 Biogas Research Center and Department of Thematic Studies – Environmental Change, Linköping University, Linkoping, SE-581 83 Sweden; 6 Department of Marine Sciences, Institute of the Sea, Av Carvalho de Mendonça, 144- Vila Belmiro, Santos-SP, Multiuser Unit of Environmental Analysis, Federal University of Rio de Janeiro, Rio de Janeiro, Brazil; 7 Environmental Science Engineering, Southern Cross University, GeoScience, Lismore, NSW Australia; 8 Department of Geography, Graduate Program in Geography, State University of Rio de Janeiro (UERJ-FFP), Rua Dr. Francisco Portela, 1470 São Gonçalo, 24435-005 Rio de Janeiro, Brazil; 9 Institut de Recherche pour le Développement (IRD), Geosciences Environnement Toulouse (GET), UMR 5563 Toulouse, France; 10 School of Geography, Earth and Environmental Sciences, and Birmingham Institute of Forest Research (BIFoR), University of Birmingham, Edgbaston, Birmingham, B15 2TT UK; 11 Department of Environmental Sciences, University of Toledo, Toledo, OH 43606 USA; 12 Departament of Meteorology, Geosciences Institute, Federal University of Rio de Janeiro, 21941-916 Rio de Janeiro, Brazil; 13 Instituto Dom Luiz, Faculdade de Ciências, Universidade de Lisboa, 1749-016 Lisbon, Portugal; 14 Forest Research Centre, School of Agriculture, University of Lisbon, 1349-017 Lisbon, Portugal; 15 Department of Geoenvironmental Analysis, Fluminense Federal University, Niterói, Brazil

**Keywords:** Carbon cycle, Biogeochemistry

## Abstract

A significant proportion of carbon (C) captured by terrestrial primary production is buried in lacustrine ecosystems, which have been substantially affected by anthropogenic activities globally. However, there is a scarcity of sedimentary organic carbon (OC) accumulation information for lakes surrounded by highly productive rainforests at warm tropical latitudes, or in response to land cover and climate change. Here, we combine new data from intensive campaigns spanning 13 lakes across remote Amazonian regions with a broad literature compilation, to produce the first spatially-weighted global analysis of recent OC burial in lakes (over ~50-100-years) that integrates both biome type and forest cover. We find that humid tropical forest lake sediments are a disproportionately important global OC sink of 7.4 Tg C yr^−1^ with implications for climate change. Further, we demonstrate that temperature and forest conservation are key factors in maintaining massive organic carbon pools in tropical lacustrine sediments.

## Abstract (web summary)

Tropical forest lake sediments are global carbon sinks, representing an important implication for climate change, of which both temperature and forest conservation are key factors in maintaining the carbon burial mechanism in lacustrine ecosystems.

## Introduction

Forests are major global atmospheric carbon (C) sinks^1–4^ of which a significant biomass fraction is transported by water flow into lacustrine sediments^5–10^ that represent temperature-sensitive organic carbon (OC) stocks that are vulnerable to greenhouse gas conversion^11,12^. However, recent global estimates of OC burial in lakes^8^ have not considered biome type nor land uses in datasets where low-latitude lakes are severely underrepresented, leading to uncertainty in current predictions of the effects of warming on large inland water C stocks. Given this lack of information, we sought to substantially increase the representation of tropical ecosystems over the previous efforts^8,10^, and estimate recent sediment OC burial rates (~50–100 year range) in global lakes through a novel approach that integrates their areal distribution^13^ across biome types^14^ and land cover^15^. In addition, their relationship with forest cover around lakes from the largest humid tropical forest in the world (i.e., the Amazon rainforest) was assessed using more accurate spatial analysis than previously reported for extensive low-latitude floodplains^16^.

Natural lakes cover approximately 2.67 million km^2^ or 1.8% of the land surface area across nearly all climate zones^13^. Lakes within watersheds are depositional environments with generally high recent OC burial rates^8,17–19^ playing an essential role in the global carbon C cycle^5,7,20^ that is disproportionate to their relatively small area. The role of lacustrine sediments as biosphere-atmosphere C sinks may be comparable to greenhouse gas emissions from inland waters^8^. Along latitudinal gradients, previous studies have reported positive relationships between temperature and OC burial in lakes spanning polar to boreal^18^, boreal to temperate^19^, and temperate to subtropical^17^ biomes. Conversely, with the exception of temperate biomes^19,21^, these initial efforts to determine global C accumulation in lake deposits have not considered remaining land cover across the world’s biomes^17,18^ with early efforts substantially underrepresenting low-latitude ecosystems. In extensive flooded areas within humid tropical forests, higher annual temperatures could actively stimulate both OC degradation^11,12^ and accumulation^22^, increasing uncertainty about the role of temperature variations in determining sediment OC burial rates.

Here, our aim is to investigate recent global OC burial rates in lake sediments (over a ~50–100-year timeframe), integrating variations in global biomes, location in reference to natural or human-altered environments, and the relative area of forest cover near lakes within the Amazon. These results show that the conservation of the surrounding forests is positively related to the highest OC burial rates in lakes globally.

## Results and discussion

### A framework to estimate lacustrine C accumulation

In order to avoid bias due to large differences with OC accumulation estimated via other methods such as ^14^C dating and sediment traps, our dataset was restricted to rates derived from ^210^Pb dating methods, considered the most accurate method for secular dating^23^. Despite the relative ~20% decrease in the number of high-and medium-latitude lakes used in the latest review of OC burial rates^10^, our efforts resulted in a proportionately larger tropical dataset from an extensive field survey of an additional 13 remote Amazonian lakes in combination with a literature compilation spanning other 43 from humid tropical forests. Accordingly, our new approach doubled the representation of warmer tropical lakes in a humid tropical forest when compared to previously reported, allowing a better representation of the global distribution of lacustrine OC burial rates along the latitudinal gradient (see details in Methods and Supplementary Material). Then, our spatial upscaling of OC accumulation was based on the latest review of global natural lake area (i.e., not including rivers, inundated floodplains or wetlands, streams, reservoirs, and artificial ponds)^13^.

This updated dataset improves the representation of low-latitude environments for global upscaling of lake OC burial (Supplementary Table 1). Further, we analysed the potential terrestrial controls over OC accumulation in lakes by comparing OC accumulation rates in lake sediments with those of forests around the world^1,4^, and by examining the relationship between recent OC burial in Amazon floodplain lakes with C dynamics in the surrounding non-flooded forests.

### Global mapping of lake OC burial

**Fig. 1:**
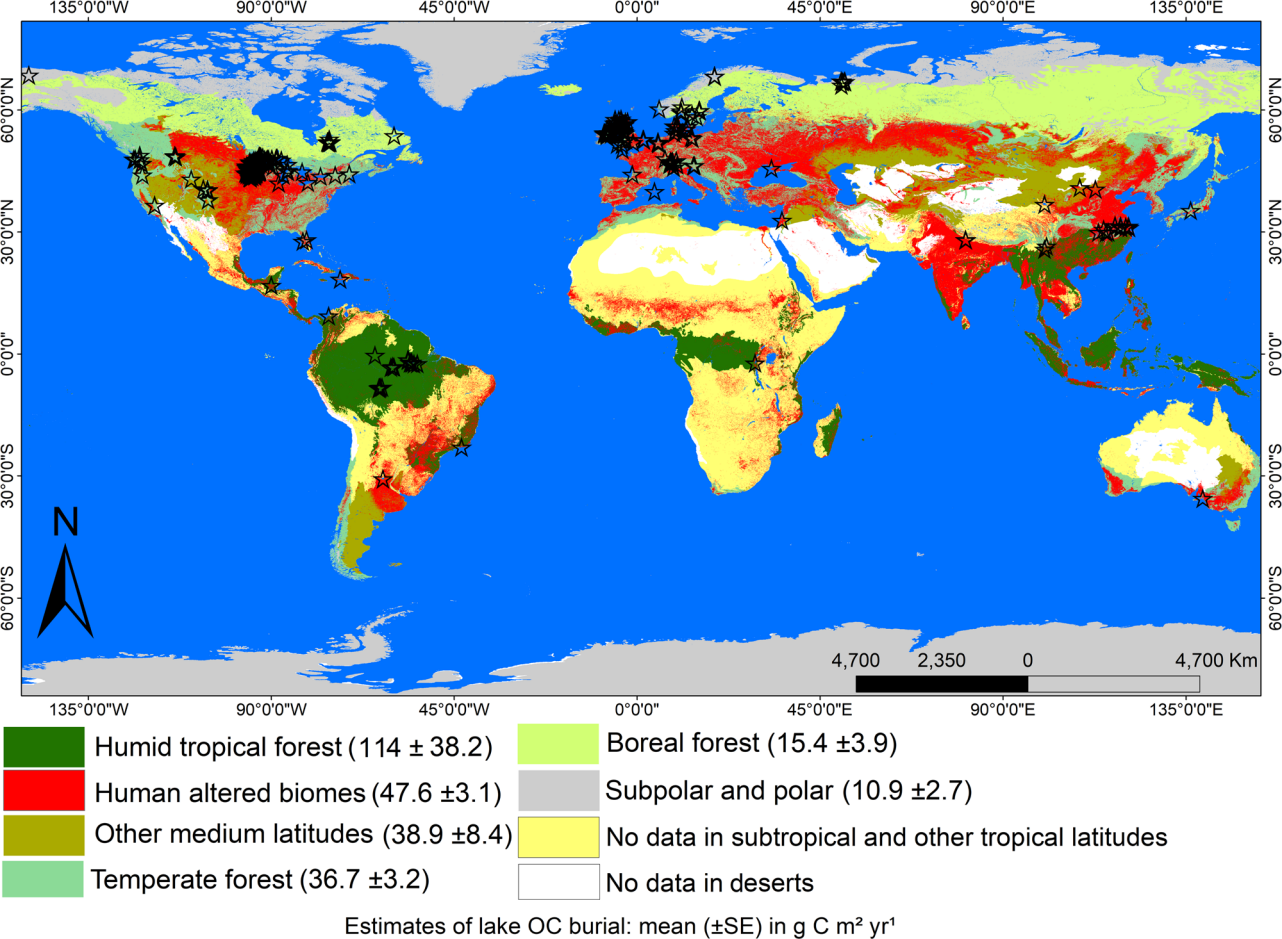
Global distribution of OC burial rates (average ± SE) in lakes across biomes. The red color indicates human-altered areas, while black stars the locations of the study lakes. No data were available for subtropical, other tropical latitudes, and deserts.

**Fig. 2:**
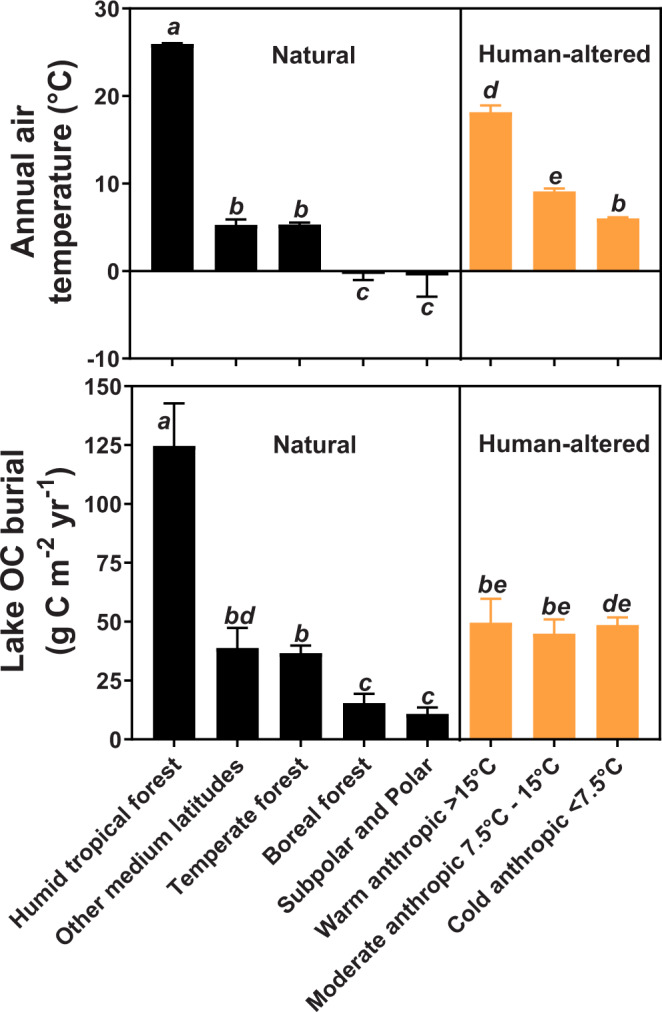
Annual air temperature and recent OC burial in lake sediments among classes of natural and human-altered biomes around the world. Classes of lake biomes were ordered from right to left along the X-axis to represent the increasing trend in the annual air temperature. The bars and error bars represent the average and standard error, respectively, and equal letters represent no statistical difference (one-way ANOVA, post hoc Tukey test, *p* < 0.05).

**Fig. 3:**
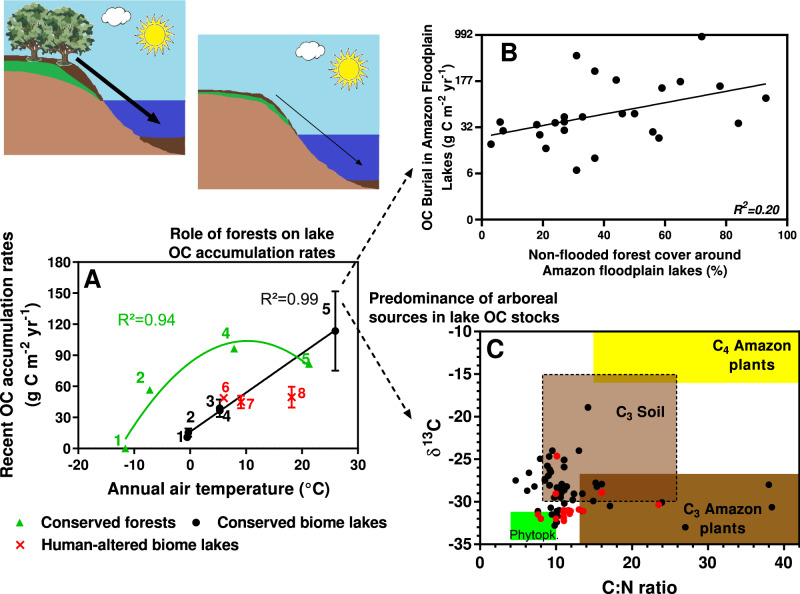
The role of forests on lake OC accumulation rates with increasing temperature. Panel **A** shows the relationships between forest OC accumulation (green triangles), lake OC burial in natural (black circles), and human-altered (red crosses) biomes with annual air temperatures along the global gradient. The number 1 represents the subpolar and polar biomes; 2 the boreal forest; 3 temperate forest; 4 the other medium latitudes; and 5 the humid tropical forest. In turn, numbers 6, 7, and 8 represent the human-altered biomes in cold, moderate-temperature, and warm anthropic environments, respectively >15.0, 7.5–15.0, and <7.5 °C. The solid green line indicates the fitted second-order polynomial model of forest OC accumulation (g C m^−2^ yr^−1^) = 82.93 ± 15.10 + 4.01 ± 1.02 × Temperature (°C) + 0.08 ± 0.02 × Temperature (°C)²; *p* < 0.01, while the solid black line represents the linear model of lake OC burial in natural biomes (g C m^−^² yr^−^¹) = 4.23 ± 0.08 × Temperature (°C) + 15.15 ± 1.01; *p* < 0.0001. All error bars in Panel **A** represent the SE. Panel **B** shows the relationship between OC burial in Amazon floodplain lakes and the relative area of non-flooded forests in their surroundings (see more details in Methods). The solid black line in this panel represents the exponential regression of Amazon lake OC burial (g C m^-^² yr^-^¹) = 56.21 ± 4.943 × e^0.01608 ±0.008 × Non-flooded forest area (%)^; *p* < 0.01 (note the logarithmic scale on the *y*-axis in panel **B**) for 6 km² buffer (See Methods for more details). Panel **C** shows the scatter plots of the δ^13^C values from the organic material fraction vs C:N molar ratios of the Amazon lake sediments based on data from our own survey (black circles, *N* = 54) and a compilation from the literature (red circles *N* = 18). The boundaries of this panel **C** are based on endmember values reported in the literature (see Methods and Supplementary Material for further details) and the relative contribution to the Amazon lake sediments are as follows: C_3_ soil (light brown rectangle, 41%), C_4_ (yellow rectangle, 0%), C_3_ (dark brown rectangle, 20%), and phytoplankton (lime green rectangle, 7%).

Our results along the latitudinal gradient demonstrate consistently high recent OC burial rates in the humid tropical forest lakes, with an average (± standard error; SE) of 113.5 (±18.1) g C m^−2^ yr^−1^. In natural biomes in temperate, boreal or polar, and subpolar latitudes OC burial rates reached 38.9 (±8.4), 36.7 (±3.2), 15.4 (±3.9), or 10.9 (±2.7) g C m^−2^ yr^−1^ respectively, while human-altered environments showed 47.6 (±3.1) g C m^−2^ yr^−1^ independent of temperature variation (Fig. 1 and Supplementary Fig. 1). The observed higher lake OC burial rates in warm low-latitudes contribute to the climate-C-burial debate in global lakes, confirming prior findings on the likelihood of warming increasing C accumulation in lacustrine sediments^17–19^. As biological remineralization and subsequent loss of large OC stocks to greenhouse gases with increasing temperature has also been reported^11,12^, our findings demonstrate that both burial and degradation of OC may be enhanced in a warming world. Increases in OC inputs^24^ and burial^18^ in lacustrine sediments have been attributed to the enhanced production of aquatic and terrestrial biomass, which reach inland waters under warmer conditions. In turn, the refractory nature of terrestrial organic matter (OM)^25,26^, coupled with high oxygen consumption in bottom waters and surface aquatic sediments in lakes surrounded by tropical forests^27^ typically leads to greater preservation of the OC pool in flooded areas^26^. Significantly higher lake OC burial with increasing annual air temperature (*p* < 0.05, one-way ANOVA followed by the Tukey’s post hoc test; Fig. 2) and the resulting positive linear regression between both variables were observed only in natural biomes (*p* < 0.05; Fig. 3A) a trend that confirms prior regional studies at high and mid-latitudes^17–19^.

Consistent with this, humid tropical forest lakes showed an average OC accumulation of ~23 and 8% higher than eutrophic and hypereutrophic lakes at medium and high latitudes reported by Anderson et al.^28^. (see Supplementary Discussion for more details). This suggests that eutrophication and subsequent water contamination could be insufficient to offset reductions in recent OC burial in lakes surrounded by lower forest cover in tropical areas, even with the expected short-term increases of biomass inputs to inland waters following deforestation in the watershed^21^. Although the relationship between long-term forest productivity and terrestrial C export to the aquatic ecosystem are well known^24^, there are few studies assessing the effects of forest cover on OC burial in humid tropical lakes^29^.

Our results indicated that warmer lakes had average OC accumulation rates ~3-fold higher in the Amazon Forest than tropical lakes under intense eutrophication in urban or cropland areas (366.2 ± 51.0 and 129.5 ± 12.9 respectively, *N* = 5 for each). Considering the observed relationship between lake OC burial and temperature (Fig. 3A), we estimated that recent C accumulation rates in warm lakes at human-altered biomes here (>18 °C) could be only ~50% of those predicted in conserved areas, while the biomass sink in cold lakes were less sensitive to human alteration (~6 and 9 °C). This supports an integrative perspective on lake OC burial debate, in which both climate^2,8,11,12,17,18,20,26,30,31^ and land cover^3,19,21,29^ might control vast C stocks in lacustrine sediments around the world.

The more intense OC burial in humid tropical forest lakes is due to the large organic inputs from highly productive terrestrial vegetation^1^ into flooded areas of this biome^32^ which fuel intense C cycling processes^11,22^. The role of rainforests in promoting intense OC accumulation in warm lake sediments at low latitudes is also independently demonstrated by the positive linear regression between recent OC burial rates in Amazon lakes and the relative area of non-flooded forest cover in their surroundings (*p* < 0.05, Fig. 3; see details in Methods and Supplementary Material). Considering the δ^13^C values and C:N molar ratios for the major OM sources documented for the Amazon forest^25^, both C_3_ soil and woody plants accounted for over 60% of the OM source to Amazon lake surface sediments (Fig. 3C). This higher participation of arboreal sources in lake OC burial might still be conservative since algae and C_4_ plants are more susceptible to mineralization before burying in deeper layers of aquatic sediments^22,29^ (see more details in Supplementary Discussion).

### Tropical drivers of OC accumulation in lakes

Our data demonstrate that humid tropical Amazon forest lakes are burying approximately 15% more OC per unit area than previously reported^1,4^ (Fig. 2), indicating that local lakes are destinations for significant proportions of the OM produced in the forest. The variation of recent OC burial rates in humid tropical forest lakes reached a SE of ±38.35 g m^−2^ yr^−1^, which was from 4- to 14- fold higher than those observed in other natural and human-altered biomes. This finding demonstrates warmer tropical lakes contain a broader range of processes involved in C cycling (i.e., greater both C uptake and release rates) than those at higher latitudes^31^. In contrast to the much higher average OC burial rates we observed in natural forest areas, the intense mineralization rates under higher annual temperatures^11,12^ and the lateral export to rivers^33^ have been considered important processes to reduce C accumulation in lakes after long-term deforestation in the humid tropics (i.e., declines in terrestrial OM sources)^29^. Specifically, our study design supports conclusions about the potential role of humid tropical forests on global C stocks in lacustrine sediments. Indeed, previous evidence has reported spikes of lake OC burial following the removal of forest in the watershed but clearly restricted to the initial period of logging in riparian regions^29^ (see Supplementary Discussion in Supplementary Material for more details). These contrasting processes of intense increases and decreases in inputs of terrestrial biomass under warm tropical conditions are therefore likely to contribute to the observed variability expressed as coefficient of variation (CV) or SE (Figs. 2, 3 and Supplementary Table 1) of OC burial in tropical lakes.

Overall, rates of recent OC burial observed in humid tropical forest lakes were among the highest in the world and comparable to other known productive ecosystems, within the same order of magnitude as those reported in seagrass meadows and saltmarshes or up to two times higher than in mangroves^34^. Even assuming a much smaller area than other biomes (~3% of the global lake area. See details in Methods and Supplementary Material), we found that humid tropical forest lacustrine sediments represent a disproportionately large C sink that accounts for ~10% of the annual C accumulation in lakes globally (Supplementary Table 2).

Applying our recent OC burial rates to global lake area^13^ resulted in an estimated global sink of 80 Tg C yr^−1^, which is equivalent to ~27% of estimated global carbon dioxide (CO_2_) emissions from lake waters to the atmosphere (i.e., 292 Tg C yr^−1^)^20^. This estimate for tropical natural forest lakes may be an underestimate as we do not include data for calculating the contribution of all tropical and subtropical lakes (see details in Methods and Supplementary Material). The C sequestration rates in sediments may be even more important relative to atmospheric emissions, given that CO_2_ concentrations and subsequent air-lake fluxes have been overestimated by the widespread use of the method based on pH and alkalinity in previous global reviews for freshwaters^35^.

Although still, an underestimate at the global scale, by using conservative estimates of tropical lake areas (which do not consider small lakes), our weighted average per lake area unit (29.70 ± 5.30 g m^−2^ yr^−1^; Supplementary Table 1) is ~20 and 50–70% higher than previously reported^8,10^. These higher values are likely due to our study increasing the representation of previously neglected tropical ecosystems. However, our estimate is still ~30% lower than a prior upscaled estimate of lacustrine OC burial^8^, which we attribute to their use of a global lake area that is ~85% higher than in the most recent review^10^. We used a more realistic natural lake area estimate from Messager et al^13^ than was used in previous estimates of global lake OC burial^8,10^, which would otherwise have resulted in a large overestimation of the lacustrine area through the inclusion of artificial or non-lake waters^36^. For the area estimated from Messager et al^13^ area estimate, lake polygons were identified from a large compilation of global data sources, followed by the replacement of those that were either erroneous or inaccurate.

### Uncertainties and implications

Although more studies on intra-and inter-ecosystem heterogeneity and low-latitude environments are still required, our expanded dataset clearly demonstrates that previously unaccounted for lake sediments and land cover exerts globally significant control over ecosystem C balances. In particular, the results here demonstrate the importance of forests in determining global lacustrine OC accumulation, identifying the need for both climate and land-use changes to be considered integrated drivers of vast C stocks in lacustrine sediments in a warming world. Indeed, our findings suggest an aquatic component of the C cycle that until now has been little examined and is highly sensitive to reduced forest cover.

The long-term decline of OC inputs due to logging^29^ or physiological constraints on primary production due to climate change (i.e., “savannization”)^3^ could further add to the expected decline in lake C stocks through enhanced biological OM mineralization under higher temperatures^11,12^. Such deforestation-related mechanisms could lead to reductions or even negations of lake C burial that had been widely predicted under existing global warming scenarios^8,17–19^. These effects are most pronounced in the tropics, which account for around half of all increases in global net primary production (NEP) despite being only around 22% of the land area, highlighting the need to preserve tropical ecosystems of which forests, and the lakes they contain, merit specific attention^30^.

Given the need to limit climate change to within 1.5 degrees C of warming this Century, these newly-identified mechanisms of aquatic C loss in humid tropical forests present us with both a challenge and opportunity in tackling climate change. A challenge is that humid tropical forests are particularly vulnerable to ongoing regional land-use change^37^; and an opportunity in that the immediate implementation of conservation and afforestation programs focused on extensive tropical areas rich in lakes will likely arrest ongoing lake sediment C loss while restarting their globally important C sequestration function.

## Methods

### Sources and calculation of OC accumulation rates

A literature review and a wide field survey were conducted with the objective of finding direct measurements of recent OC burial rates, with major emphasis given to the inclusion of underrepresented tropical lakes. A cross-reference search was performed using “Web of Science”, “Science Direct”, “Google Scholar”, and Ph.D. thesis platforms until December 2019. We created a dataset (*N* = 397 lakes) from published literature, including three reviews^28,38,39^, 21 other papers, and three thesis (see all data and sources in the *work dataset*), which were combined with new records from our field surveys in 2014, 2015, and 2016 (*n* = 13) in a wide area of 580,000 km^2^ along a maximum distance of 1170 km (Supplementary Fig. 3, and *work dataset*). This approach increased by ~75^10^ and 130%^8^ the number of tropical forests that are represented over previously published studies.

All data were based on estimations of OC burial using profiles of ^210^Pb, excluding other methodologies. Then, we provide data regarding the sediment OC mass accumulation rates. The ^210^Pb was measured by Alpha or Gamma-ray activity. ^226^Ra was measured through Gamma-ray and activities were determined through daughter peaks of ^214^Pb and ^214^Bi. The excess ^210^Pb (^210^Pb_ex_) activity is estimated by subtracting the ^226^Ra from the total ^210^Pb activity. The total ^210^Pb activity decay and ^210^Pb_ex_ were used to date sediment intervals through the constant rate supply (CRS), and constant initial concentration (CIC) models^23,40^ as the difference between both is negligible over the assessed timeframe (50–100 years)^41^. The CRS model determined ~77% of the presented OC burial rates, and we used the CRS model for the sediment cores sampled here (Supplementary Fig. 4). Moreover, to avoid bias towards time periods with higher resolution, the ^210^Pb measurements were evenly distributed along the sediment profile. Then, the sediment mass accumulation rate (SAR) was determined as the product of sediment accumulation (cm yr^−1^) and respective dry bulk density [g cm^−3^] in each interval^42,43^. The results are expressed as SAR [cm year^−1^], dry bulk density [g cm^−3^], and OC content (OC Burial [g C m^−2^ y^−1^] = SAR [cm year^−1^] × dry bulk density [g cm^−3^] × [% OC/100]).

The whole-lake OC burial was then estimated by three alternative methods depending on the available data as described by Mendonça et al. (2017): (1) the average of such rates derived from two or more sediment cores sampled at different parts of a given ecosystem; (2) dividing the rate from a single core sampled in the preferential sediment deposition area by the sediment focusing factor (SFF) following Blais and Kalff (1995) when mean lake slope is measured; or (3) dividing the single rate by the SFF reported into groups according to lake maximum depth (≤5 m, >5–10 m, >10–30 m, >30–90 m, and >90 m) when bathymetric data were not determined.

Concerning forest OC accumulation, we compiled the estimates of each compartment reported in a previous review:^1^ Soil Organic Matter, Living Biomass, Deadwood, Litter, and Wood product (Supplementary Table 2). The equatorial carbon in soil was not estimated by Pan et al. (2011), but we obtained it from Malhi and Grace (2000). Then, we also used the forest area inventory provided in Pan et al. (2011) but subtracted the tropical forest wetland area reported in Mitra et al.^44^. This approach based on the comparison of relatively longer periods (around 50–100 years) with forest OC accumulation rates (around 10 years) allowed more conservative estimates of lake OC burial, as non-mineralized fractions in terrestrial soils may continue to decay over time (i.e., resulting in an overestimation of these rates in the forest as related to the lake sediment).

### Our survey and chemical analyses

Thirteen sediment cores (approximately 50–90 cm long; Supplementary Fig. 4) were sampled from the deepest area of each lake using a gravity core. The sediment cores were then sealed and cooled for transport. In the laboratory, cores were sectioned at 2 cm intervals; samples with known volumes were weighed and freeze-dried. The dry bulk density was calculated by dividing the dry weight of the sediment by the initial volume^45^. The total organic carbon (total C_org_) and the δ^13^C and δ^15^N isotopic compositions (ground to powder samples) were analyzed in a Flash Element Analyzer coupled to a Thermo Fisher isotope ratio (Delta V IRMS) mass spectrometer. The analytical precision is: C = 0.1%, N = 0.1%, δ^13^C = 0.1‰, and δ^15^N = 0.15‰. The CRS model (^210^Pb dating) was applied using all ^210^Pb measurements in each sediment core collected for the present study. All Amazon lakes here were situated in conserved areas during sampling (2007–2012), with negligible land-use change along a natural gradient of non-flooded forest cover in the immediate surroundings. In this way, sediment profiles of these lakes maintained relatively high OC burial rates over ~50–100 years (Supplementary Fig. 4), with no clear pattern of change or increases due to the potential influence of terrestrial OM inputs (Fig. 3). The scatter plot of δ^13^C and C:N ratio of Amazon lake surface sediments (0-6 cm samples) are the result of our survey and published data (*N* = 18 and 54 lakes, respectively), following the boundaries of major OM sources^46–49^.

### GIS dataset

We used the HydroLAKES database^13^ to determine natural lake areas around the world (see Supplementary Table 1). This model shows a total surface lake area of 2.67 × 10^6^ km^2^, which was created by compiling, correcting, and unifying several near-global and regional datasets. The resulting map scale was estimated to be between 1:100,000 and 1:250,000 for most lakes globally, with some coarser ones at 1:1 million. Each lake in this database was classified according to the respective biome.

Further, the seven categories of terrestrial world biomes here (i.e., (1) Humid tropical forest; (2) Other tropical and subtropical latitudes; (3) Temperate forest; (4) Boreal forest; (5) Other medium latitudes; (6) Subpolar and polar latitudes; and (7) Deserts) were extracted from a shape previously published^14^ (available at https://www.worldwildlife.org/biome-categories/terrestrial-ecoregions). In turn, we used data from the Land Cover (GLCNMO)—Global version^15^ (available at https://globalmaps.github.io/glcnmo.html) to separate human-altered areas (i.e., grouping together cropland, urban, and paddy field) from those natural, which were still subdivided into three more classes according to the annual air temperature of each lake (<7.5, 7.5–15.0, and >15 °C; Supplementary Fig. 3). Temperature data was extracted from the Atlas of the Biosphere dataset, using the model from the *Climate Research Unit/University of East Anglia* (available at https://nelson.wisc.edu/sage/index.php). The resulting ten classes were obtained by GIS overlay analysis of biome types for natural areas or classes of annual air temperature (i.e., cold, intermediate, and warm) for human-altered areas (see details on decision tree in Supplementary Fig. 5). Thus, each study lake was classified among either the following natural biomes: (1) Humid tropical forest (*n* = 44); (2) Other tropical/subtropical latitude ecosystems other than forest (no data); (3) Temperate forest (*n* = 161); (4) Boreal forest (*n* = 25); (5) Other mid-latitude ecosystems other than forest (*n* = 16) and (6) Subpolar and polar latitudes (*n* = 09); and (7) Deserts (no data) or human-altered biomes: (8) Cold anthropic (*n* = 90), (9) Moderate anthropic (*n* = 29), and (10) Warm anthropic (*n* = 24).

In relation to Amazon lakes here (Fig. 3B), we used ASAR global monitoring mode image (available at https://earth.esa.int/handbooks/asar/CNTR6-2-5.html) to determine flooded and non-flooded areas around each studied ecosystem. Then, non-flooded areas were reclassified into two categories (1) forest and (2) natural and anthropic field cover, using MODIS/Terra Surface Reflectance 8-Day L3 Global 500 m SIN Grid V006 (available at https://lpdaac.usgs.gov/dataset_discovery/modis/modis_products_table/mod09a1_v006. Moreover, we selected MODIS and ASAR products from periods when the water level of the main river of each study Amazon study lake was closest to the 23-year average (deviation of until ±8%; Supplementary Table 3), obtained from the full Brazilian National Water Agency dataset (available at http://www.snirh.gov.br/hidroweb/publico/medicoes_historicas_abas.jsf). To perform the segmentation process of ASAR and MODIS products, an object-oriented classification method was implemented (see details on decision tree is in Supplementary Fig. 6), using a multiresolution algorithm with scale parameters 10, and 0.2 of form and 0.8 of compactness. Finally, the relative area of non-flooded forest was determined in different sized buffers (3, 4, 6, 8, 12, and 18 km^2^) around a given lake at the same river floodplain (i.e., excluding the main river or its other margin). This approach using buffers in ASAR and MODIS products is more appropriate in order to assess the potential catchment and terrestrial input sources into lakes situated in flat areas, such as inside the Amazon Forest where the immediate watershed cannot be delimited due to the low altitude. After analysing each sized buffer (Supplementary Fig. 7), we found that the 6 km^2^ area provided the best-fit relationship between recent OC burial in Amazon floodplain lakes and non-flooded forest area (higher r² of linear regression, *p* < 0.01; Supplementary Fig. 2). These results allowed us to identify trends of C accumulation in lacustrine sediments with non-flooded forest cover in the surrounding area.

### Comparisons with global lake C emission

Raymond et al. (2013) consider reservoirs were treated as similar to natural lakes because their *p*CO_2_ has been shown to be elevated only during the initial ~15 years after impoundment. This paper estimates a global lake and reservoir surface area of 3,000,000 km^2^, of which 91.3% is lakes and 8.7% is reservoirs. Thus, we calculate 91.3% of 320 Tg C yr^−1^, around ~27% (or ~292 Tg C Yr^−1^) of our estimates.

### Statistics

The OC burial log-transformed data met the assumptions of parametric tests^50^; the data were not normally distributed (Kolmogorov–Smirnov, *p* < 0.05) and variances were heterogeneous (Bartlett, *p* < 0.05). Mean (Supplementary Table 4) and parametric tests were used to compare sampling groups (Kruskal–Wallis, *p* < 0.05). The same tests were performed in relation to temperature; however, in this case, the data were not log-transformed. Thus, we used one-way ANOVA followed by a Tukey post-test (significance *p* < 0.05) to compare conserved and human-altered biomes. The stabilization of variance with the number of measurements randomly selected was reached (Supplementary Fig. 8).

## Supplementary information

Supplementary information

Supplementary dataset

## Data Availability

The data that support the findings of this study are available in the Supplementary Data.
