# Author Correction: Tropical forests as drivers of lake carbon burial

**DOI:** 10.1038/s41467-023-39006-2

**Published:** 2023-06-06

**Authors:** Leonardo Amora-Nogueira, Christian J. Sanders, Alex Enrich-Prast, Luciana Silva Monteiro Sanders, Rodrigo Coutinho Abuchacra, Patricia F. Moreira-Turcq, Renato Campello Cordeiro, Vincent Gauci, Luciane Silva Moreira, Fausto Machado-Silva, Renata Libonati, Thairiny Fonseca, Cristiane Nunes Francisco, Humberto Marotta

**Affiliations:** 1 Ecosystems and Global Change Laboratory (LEMG-UFF), International Laboratory of Global Change (LINCGlobal), Biomass and Water Management Research Center (NAB-UFF), Niterói, Brazil; 2 Physical Geography Laboratory (LAGEF-UFF), Department of Geography, Graduate Program in Geography, Fluminense Federal University (UFF), Niterói, Brazil; 3 Graduate Program in Geosciences (Environmental Geochemistry), Fluminense Federal University, Niterói, Brazil; 4 National Marine Science Centre, Faculty of Science and Engineering, Southern Cross University, PO Box 4321, Coffs Harbour, 2450 NSW Australia; 5 Biogas Research Center and Department of Thematic Studies – Environmental Change, Linköping University, Linkoping, SE-581 83 Sweden; 6 Department of Marine Sciences, Institute of the Sea, Av Carvalho de Mendonça, 144- Vila Belmiro, Santos-SP, Multiuser Unit of Environmental Analysis, Federal University of Rio de Janeiro, Rio de Janeiro, Brazil; 7 Environmental Science Engineering, Southern Cross University, GeoScience, Lismore, NSW Australia; 8 Department of Geography, Graduate Program in Geography, State University of Rio de Janeiro (UERJ-FFP), Rua Dr. Francisco Portela, 1470 São Gonçalo, 24435-005 Rio de Janeiro, Brazil; 9 Institut de Recherche pour le Développement (IRD), Geosciences Environnement Toulouse (GET), UMR 5563 Toulouse, France; 10 School of Geography, Earth and Environmental Sciences, and Birmingham Institute of Forest Research (BIFoR), University of Birmingham, Edgbaston, Birmingham, B15 2TT UK; 11 Department of Environmental Sciences, University of Toledo, Toledo, OH 43606 USA; 12 Departament of Meteorology, Geosciences Institute, Federal University of Rio de Janeiro, 21941-916 Rio de Janeiro, Brazil; 13 Instituto Dom Luiz, Faculdade de Ciências, Universidade de Lisboa, 1749-016 Lisbon, Portugal; 14 Forest Research Centre, School of Agriculture, University of Lisbon, 1349-017 Lisbon, Portugal; 15 Department of Geoenvironmental Analysis, Fluminense Federal University, Niterói, Brazil

**Keywords:** Carbon cycle, Biogeochemistry

Correction to: *Nature Communications* 10.1038/s41467-022-31258-8, published online 13 July 2022

The original version of this article contained an error in the Abstract, which incorrectly read.

‘We find that humid tropical forest lake sediments are a disproportionately important global OC sink of ~80 Tg C yr^−1^ with implications for climate change.’

The correct version replaces this sentence with

‘We find that humid tropical forest lake sediments are a disproportionately important global OC sink of ~7.4 Tg C yr^−1^ with implications for climate change.’

The original version of this Article contained an error in the ‘Tropical drivers of OC accumulation in lakes’ section, which incorrectly read.

‘Applying our recent OC burial rates to global lake area^13^ resulted in an estimated global sink of 79 Tg C yr^−1^, which is equivalent to ~27% of estimated global carbon dioxide (CO_2_) emissions from lake waters to the atmosphere (i.e., 292 Tg C yr^−1^)^20^’

The correct version replaces this sentence with

‘Applying our recent OC burial rates to global lake area^13^ resulted in an estimated global sink of 80 Tg C yr^−1^, which is equivalent to ~27% of estimated global carbon dioxide (CO_2_) emissions from lake waters to the atmosphere (i.e., 292 Tg C yr^−1^)^20^’

This has been corrected in both the PDF and HTML versions of the article.

