# Supplementary material for: Tropical forests as drivers of lake carbon burial
Source: Nat Commun. 2022 Jul 13;13:4051. doi: 10.1038/s41467-022-31258-8 (PMC9279284; doi:10.1038/s41467-022-31258-8)
Supplement: Supplementary file 1 — Supplementary information [file 41467_2022_31258_MOESM1_ESM.docx]

**Tropical forests as drivers of lake carbon burial**

**Supplementary Material**

**Supp. Table List**

**Supp. Table 1 -** Area, OC burial rates per area, coefficient of variation of the dataset and global carbon burial rates for each biome in the present study. *The global value was obtained using the mean weighted by the sum of lake area in each biome. **OC burial dataset for this biome is not available, so we used the mean values of conserved biomes (excluding the humid tropical forest to be conservative).

|  | | **Lake area**^1^  **[km^2^ x 10^4^]** | | **OC burial per area**  **(mean *±* SE)**  **[g C m^-2^ yr^-1^]** | **Coefficient of variation OC burial**  **[%]** | **OC burial**  **(mean *±* SE)**  **[Tg yr^-1^]** |
| --- | --- | --- | --- | --- | --- | --- |
| **Natural** | *Humid Tropical Forest* | | 6.49 | 114 *± 38.20* | 159.7 | 7.4 *± 2.49* |
|  | *Other tropical/subtropical* | | 30.45 | 25.48** | -- | 7.76** |
|  | *Temperate Forest* | | 31.99 | 36.68 *± 3.15* | 109.21 | 11.74 *± 1.01* |
|  | *Boreal Forest* | | 71.89 | 15.44 *± 3.90* | 126.28 | 11.10 *± 2.80* |
|  | *Others Mid-latitudes* | | 57.34 | 38.90 *± 8.44* | 86.84 | 22.31 *± 4.84* |
|  | *Subpolar and Polar* | | 37.16 | 10.92 *± 2.71* | 74.38 | 4.06 *± 1.01* |
| **Human-altered** | *Cold (<7.5 °C)* | | 9.71 | 48.60 *± 3.25* | 65.76 | 4.72 *± 0.32* |
|  | *Moderate (7.5 - 15 °C)* | | 14.79 | 44.89 *± 6.08* | 72.93 | 6.64 *± 0.90* |
|  | *Warm (>15°C)* | | 7.17 | 49.62 *±10.10* | 99.49 | 3.56 *± 0.72* |
| **Global** | | | 267 | 29.70 *±5.3** | -- | 79.97 *±12.77* |

**Supp. Table 2.** C stocks per area of forest in each biome. For tropical biomes, we subtracted the flooded areas to obtain only the contribution of non-flooded forests.

|  | | **Temperate forest** | **Boreal forest** | **Humid tropical Forest** | **Global** | **Source** |
| --- | --- | --- | --- | --- | --- | --- |
| **C stock**  **[Tg C yr^-1^]** | *Biomass* | 345 | 117 | 1167.0 | 1629.0 | ^2^ |
|  | *Dead wood* | 42 | 53 | 109.0 | 204.0 | ^2^ |
|  | *Litter* | 46 | 103 | 17.0 | 166.0 | ^2^ |
|  | *Soil* | 160 | 125 | 99.9 | 384.9 | ^7,4^* |
|  | *Wood Product* | 80 | 94 | 35.0 | 209.0 | ^2^ |
|  | *SUM* | 673 | 492 | 1427.9 | 2592.9 | This study |
| **Area**  **[km^2^x10^5^]** | *Forest* | 76.70 | 113.50 | 194.90 | 385.10 | ^2^ |
|  | *Wetland* | 6.83 | 26.50 | 20.17 | 53.50 | ^5^ |
|  | *Forest-wetland* | 69.87 | 87.00 | 174.73 | 331.60 | This study |
| **C Sink**  **[g C m^2^ yr^-1^]** | *Forests-only* | 96.33 | 56.55 | 81.72 | 78.19 | This study |

* The reference 8 refers only to soil C stock in humid tropical forests.

**Supp. Table 3**. Values of main rivers water levels and acquisition date of satellite data for each Amazon floodplain lakes. We employed this data for the use and cover buffers (12km²).

| **Lake** | **Months/years of selected images** | **Water Level**  **[m]** | **Ratio**  **Average of 23 years: Monthly water level value [m]** |
| --- | --- | --- | --- |
| *Acarabixi* | 04/2005 | 17.39 | 1.02 |
| *Cristalino* | 04/2005 | 17.39 | 1.02 |
| *Calado* | 02/2003 | 11.45 | 0.98 |
| *Jacaretinga* | 02/2003 | 11.45 | 0.98 |
| *PA09 (Verde)* | 01/2005 | 5.07 | 1.03 |
| *Paca* | 12/2004 | 17.33 | 0.92 |
| *Nazaré* | 12/2004 | 17.33 | 0.92 |
| *Conceição* | 12/2004 | 17.33 | 0.92 |
| *Santa Catarina* | 12/2004 | 17.33 | 0.92 |
| *Demarcação* | 12/2004 | 17.33 | 0.92 |
| *Santa Ninha* | 08/2004 | 5.16 | 1.07 |
| *Reis* | 12/2007 | 18.63 | 0.99 |
| *Pupunha* | 12/2007 | 18.63 | 0.99 |
| *Puruzinho* | 12/2007 | 18.63 | 0.99 |
| *Ajudante* | 12/2004 | 17.33 | 0.92 |
| *Araca* | 12/2004 | 17.33 | 0.92 |
| *Batata* | 12/2004 | 17.33 | 0.92 |
| *Brasileira* | 12/2004 | 17.33 | 0.92 |
| *Carana* | 01/2005 | 5.07 | 1.03 |
| *Flexal* | 12/2004 | 17.33 | 0.92 |
| *LC1* | 08/2004 | 5.16 | 1.07 |
| *Mussurá* | 12/2004 | 17.33 | 0.92 |
| *NR15* | 04/2005 | 17.39 | 1.02 |
| *Pacoval* | 08/2004 | 5.16 | 1.07 |
| *TA1 (Joá)* | 08/2004 | 5.16 | 1.07 |
| *Tucunaré* | 12/2004 | 17.33 | 0.92 |

**Supp. Table 4**. Descriptive statistics in conserved and human-altered biomes.

| **Biomes** | **N** | **Minimum** | **Maximum** | **Mean** | **Std. Error of Mean** | **Coefficient of variation** |
| --- | --- | --- | --- | --- | --- | --- |
| Humid Tropical Forest | 43 | 6.35 | 937.3 | 114.00 | 38.35 | 172.30% |
| Other Mid-Latitudes | 16 | 1.87 | 109 | 38.90 | 8.444 | 86.84% |
| Temperate Forest | 161 | 2.715 | 224.8 | 36.68 | 3.157 | 109.21% |
| Boreal Forest | 25 | 1.09 | 74 | 15.44 | 3.90 | 126.28% |
| Subpolar and Polar | 9 | 1.30 | 25 | 10.92 | 2.71 | 74.38% |
| Warm Anthropic | 90 | 0.49 | 127 | 48.60 | 3.24 | 65.76% |
| Moderate Anthropic | 29 | 2.174 | 112.1 | 44.89 | 6.08 | 72.93% |
| Cold Anthropic | 24 | 5.728 | 174 | 49.62 | 10.08 | 99.49% |

**Supplementary Figures list**


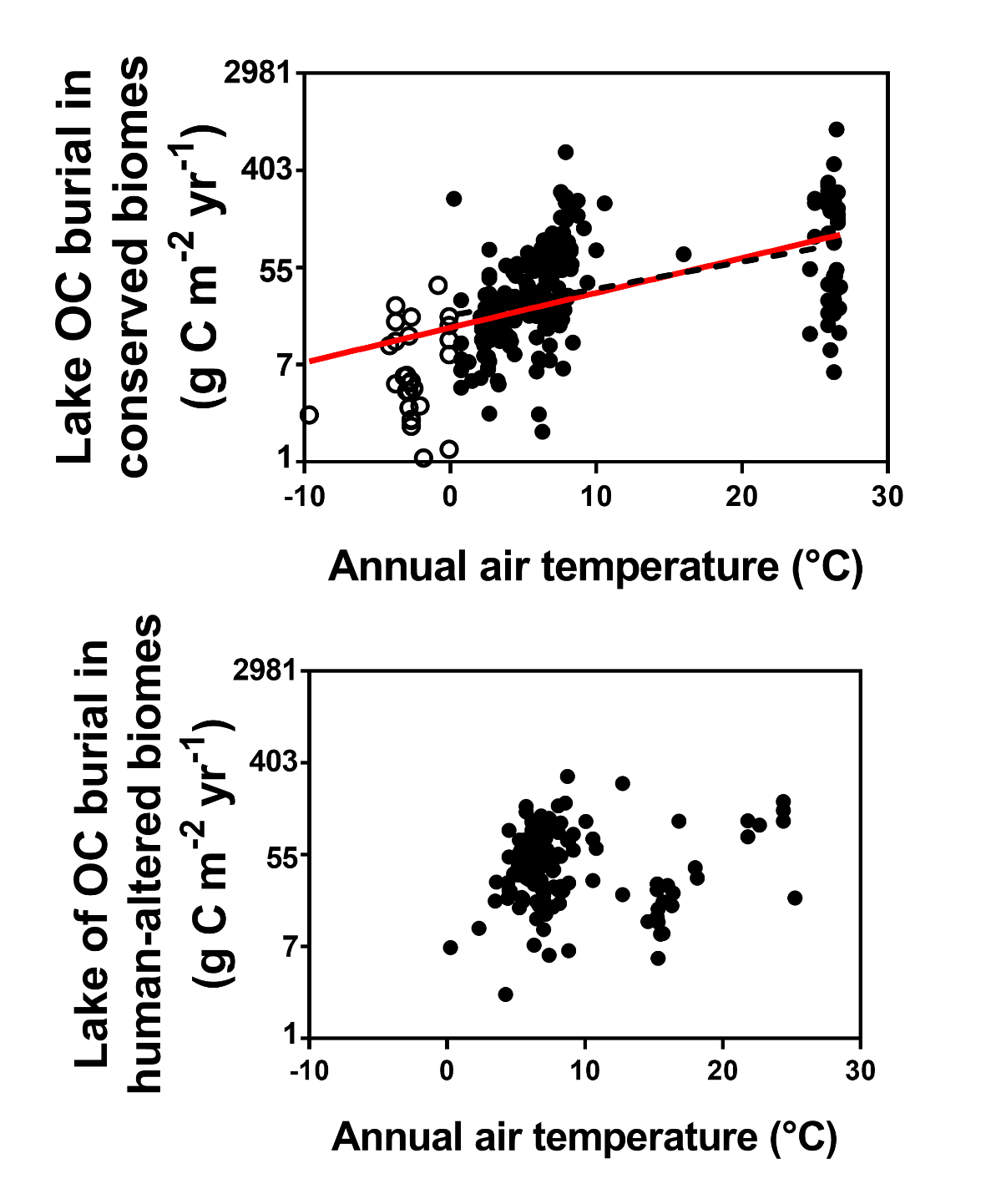


**Supp. Figure 1** – The relationship of lake OC burial rates with annual air mean temperature. Panel A shows lake OC burial and air temperature in conserved biomes; the dotted black line indicates the linear regression fit of Ln OC burial (g C m^-^² yr^-^¹) = 0.06±0.008 × Temperature (°C) + 3.02±0.09  (R² = 0.18, p<0.0001) for temperatures over 0°C (*p<0.05*); the solid red line indicates the linear regression fit of ln OC accumulation (g C m^-^² yr^-^¹) = 0.07±0.007 × Temperature (°C) + 2.764±0.09 (R² = 0.28, , p<0.0001) for all data set (*p<0.05*). Panel B shows a non-significant relationship between lake OC burial and air temperature in human-altered biomes (*p>0.05*). Filled black circles represent lakes showing air temperatures above 0°C and non-filled circles below 0°C.


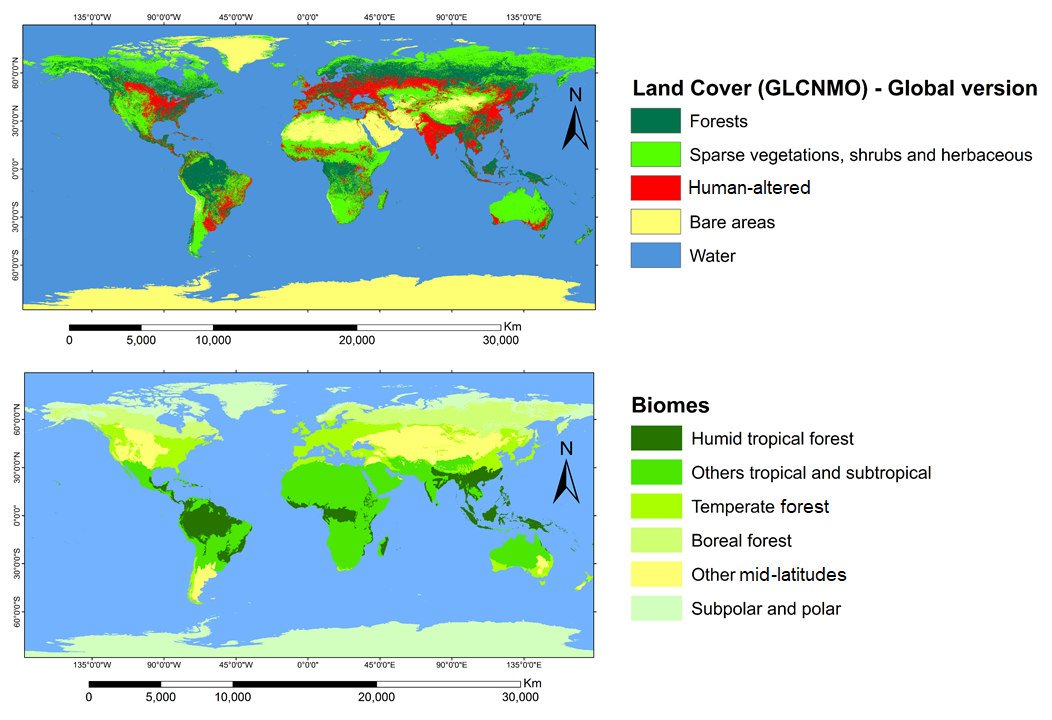


**Supp. Figure 2 –** Database used to create classes on this study. The first map shows the land use and cover map database (GLCNMO) when we extract the anthropogenic areas that were considered in this study. The second map shows the biome database and the lakes location where the OC burial data were previously compiled^6^.


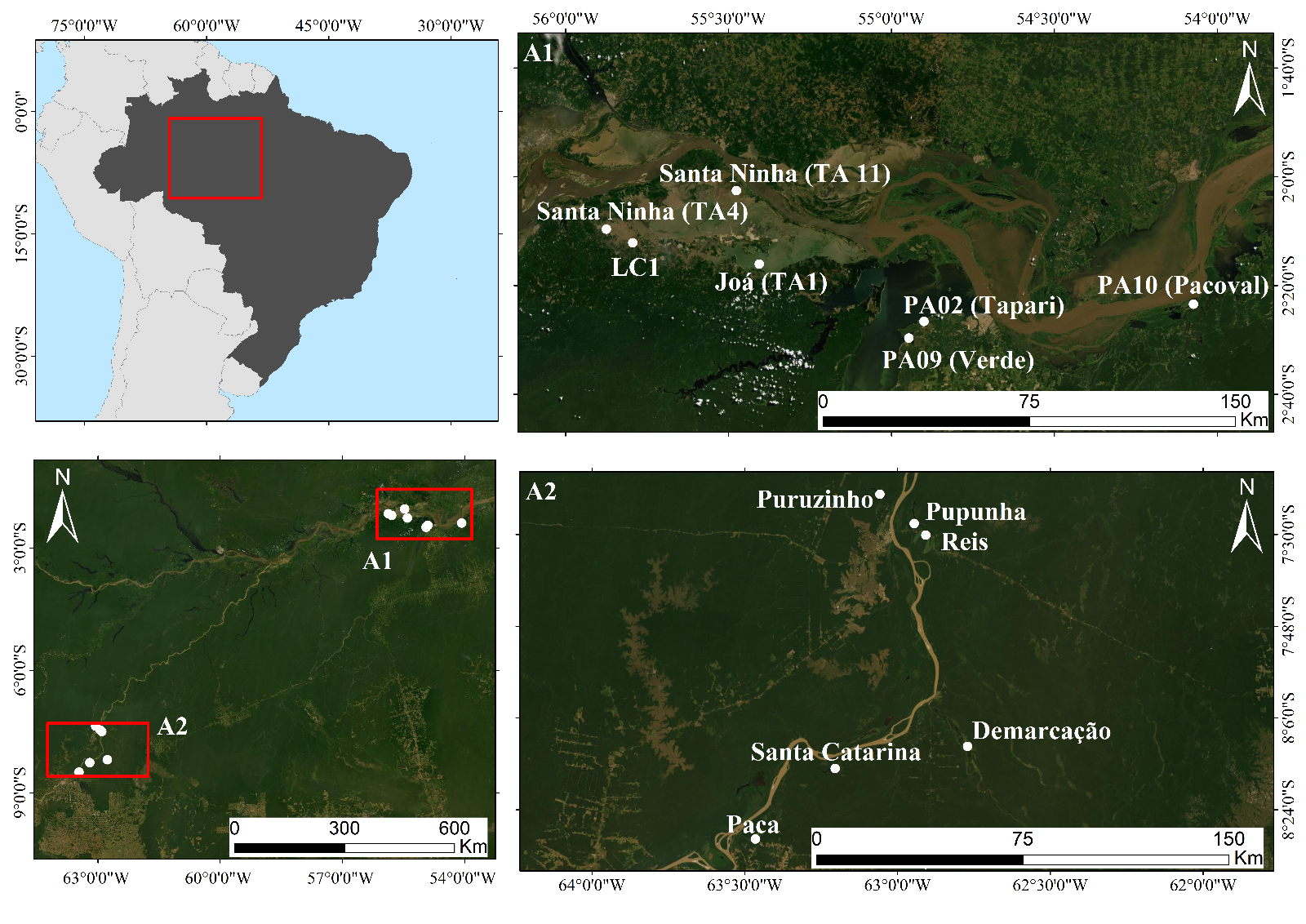


**Supp. Figure 3** – Location map of the Amazonian lakes.


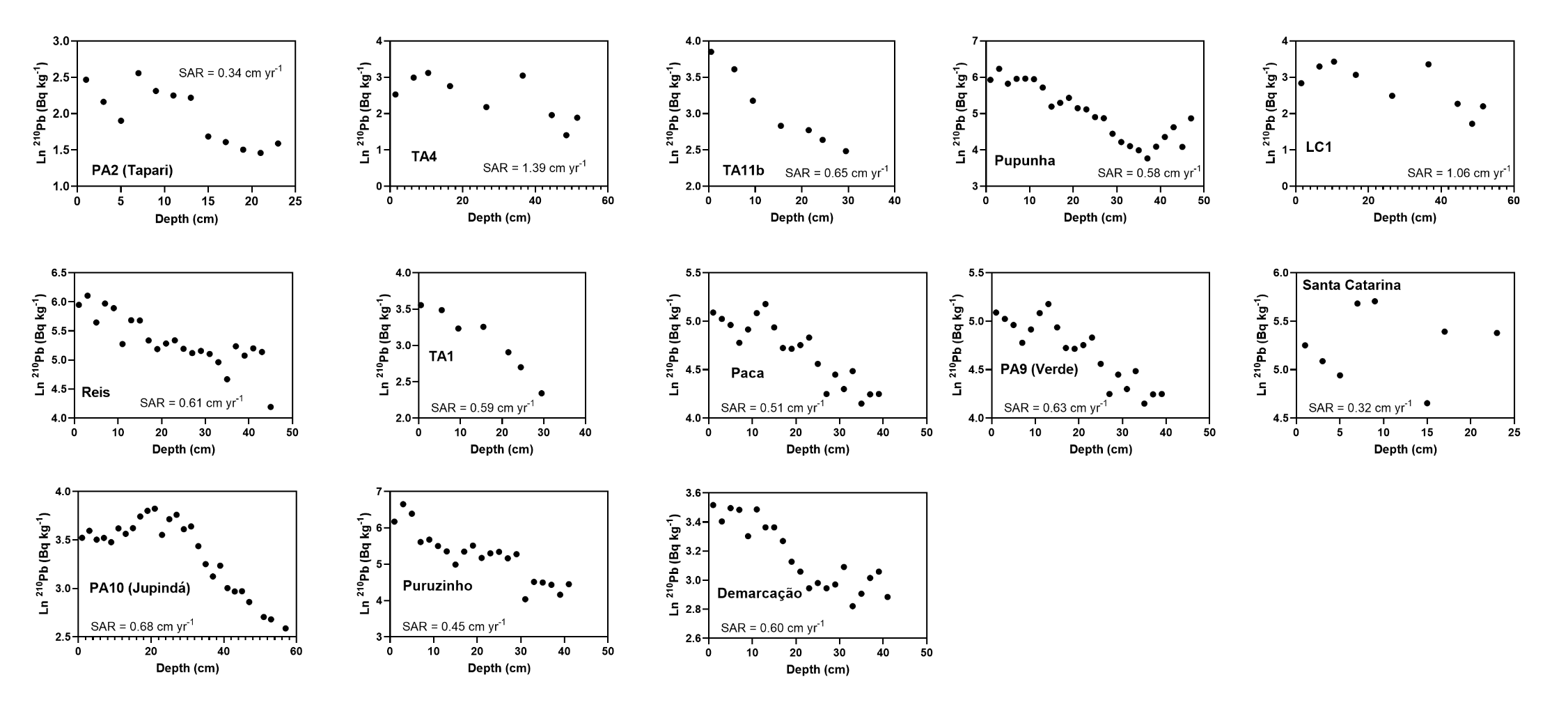


**Supp. Figure 4 -** Profiles of Ln ^210^Pb measurements and SAR from the CRS model.


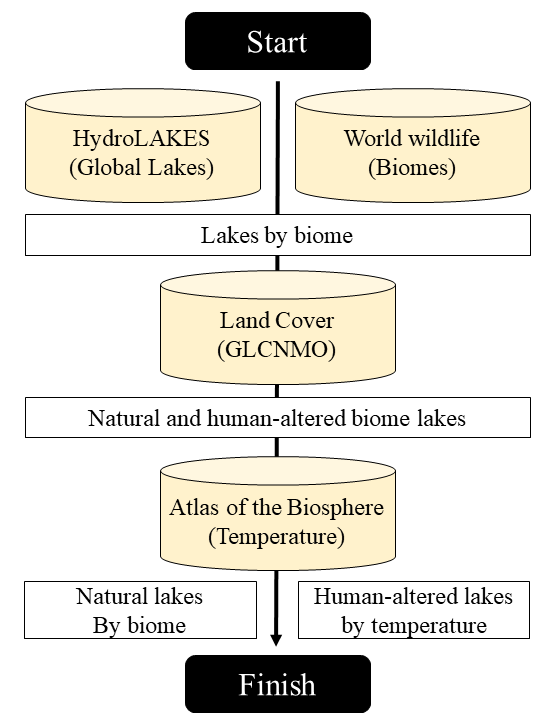


**Supp. Figure 5 -** Flowchart of the general methodology for global lakes.

**
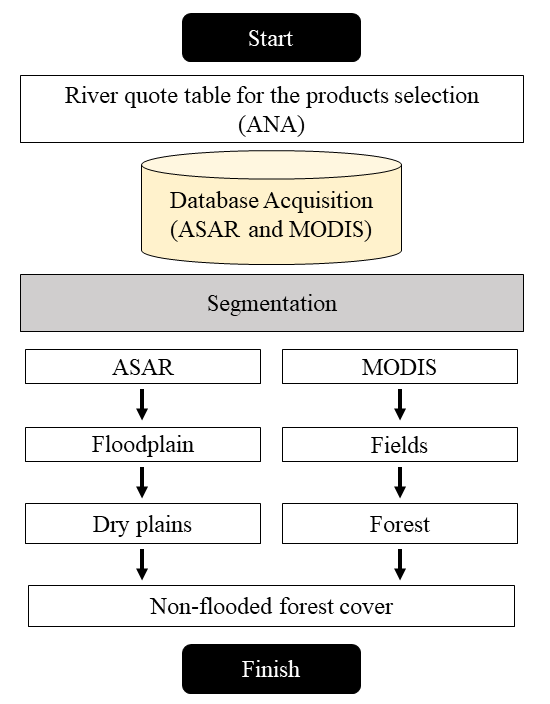
**

**Supp. Figure 6 -** Flowchart of the general methodology for amazon lakes.


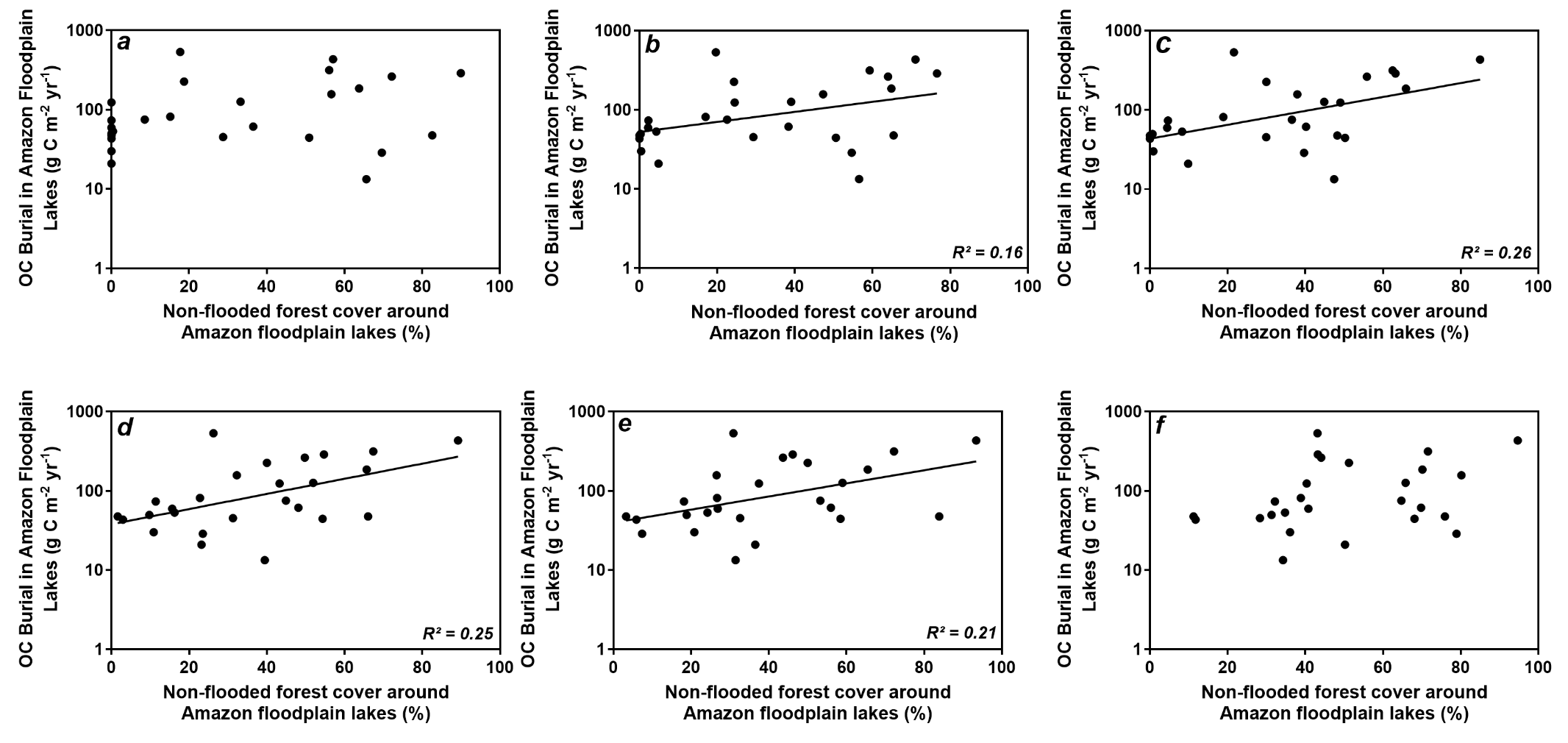


**Supp. Figure 7** – The relationship of lake Amazon floodplain OC burial rates with non-flooded forest area. The panels A, B, C, D, E and F represent size buffers of 3, 4, 6, 8, 12 and 18 km². The size of 3 (A) and 18 km² (F) were not significant (*p*>0.05). The solid black line in panels B, C, D e E represents the exponential growth model; were: B (4 Km²) is Amazon lake OC burial (g C m^-^² yr^-^¹) = 67.75±31.22 × e^0.017±0.008×Non-flooded forest area (%)^ (*p* < 0.05); C (6 Km²) is Amazon lake OC burial (g C m^-^² yr^-^¹) = 67.75±31.22 × e^0.017±0.008×Non-flooded forest area (%)^ (*p* < 0.01); D (8 Km²) is Amazon lake OC burial (g C m^-^² yr^-^¹) = 50.484±22.37 × e^0.023±0.007×Non-flooded forest area (%)^ (*p* < 0.01); E (12 Km²) is Amazon lake OC burial (g C m^-^² yr^-^¹) = 62.48±27.89 × e^0.017±0.007×Non-flooded forest area (%)^ (*p* < 0.05). Note the logarithmic scale on *y*-axis in all panels.

**
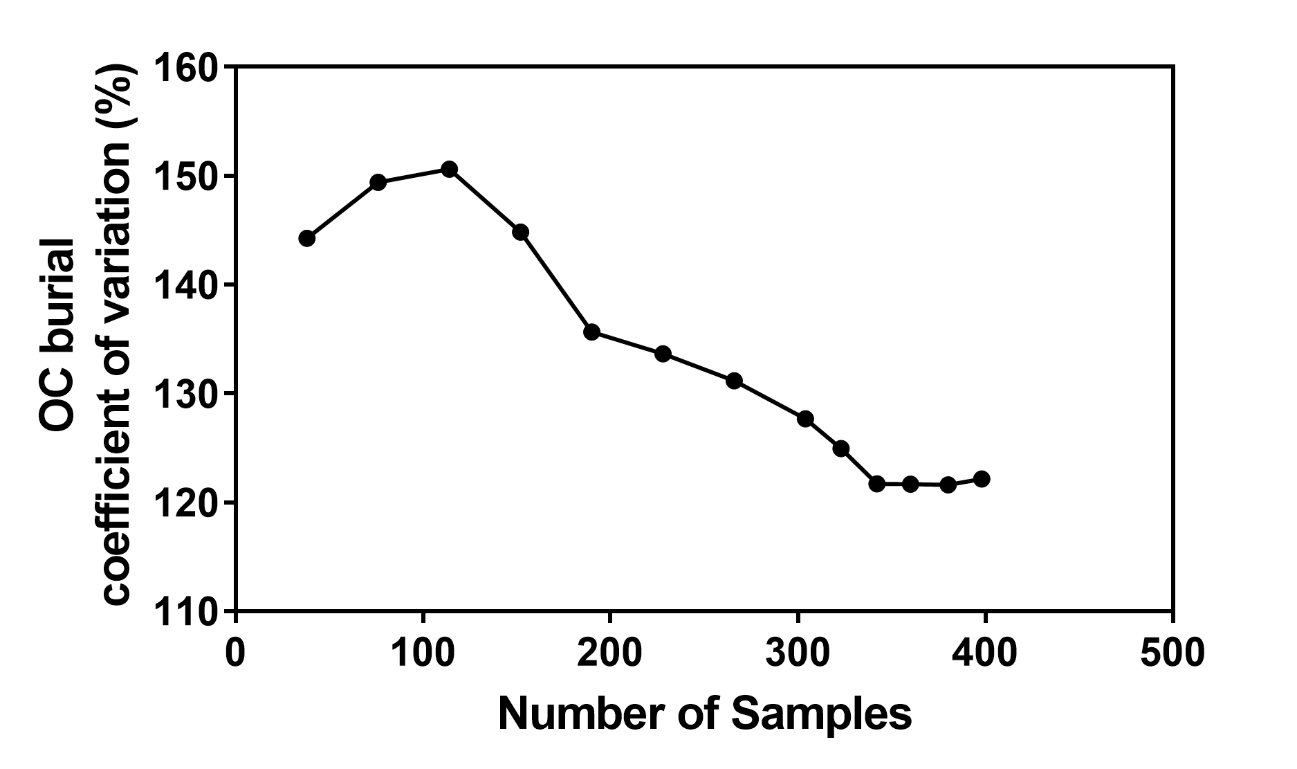
**

**Supp. Figure 8** – Relationship between coefficient of variation of OC burial rates and the randomly selected number of samples.

**
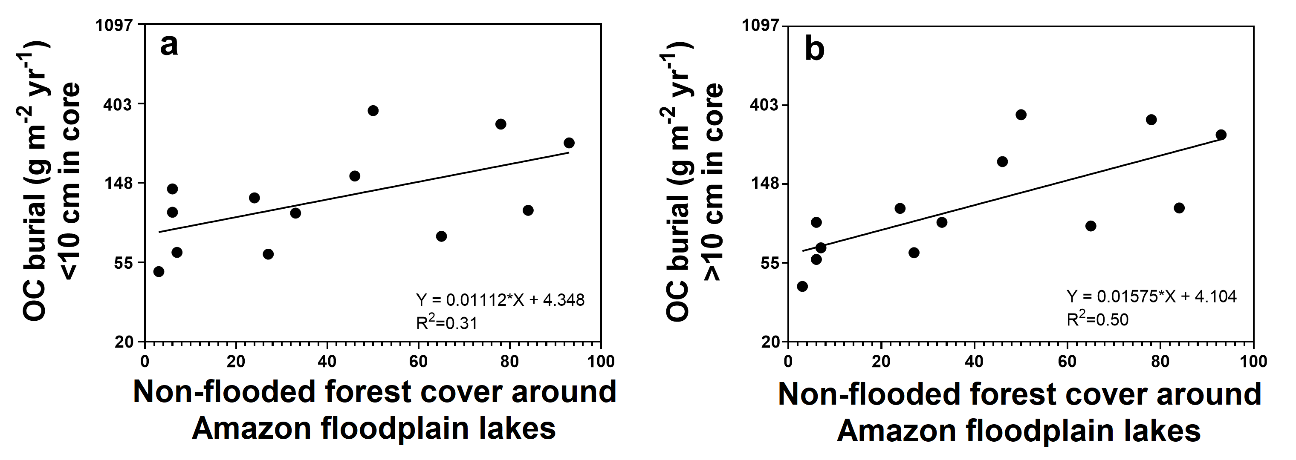
**

**Supp. Figure 9 -** Panel A show the relationship between the average above 10cm on the top of the core and the percentage of vegetation in 6 km^2^ buffer size. Panel B is the relationship between the average bellow 10cm downcore and the percentage of vegetation in 6km^2^ buffer size.

**Supplementary Discussion**

Estimates of annual OC burial in global lakes from our dataset were ~25, 13-63, 38-50 and ~10 % higher than Mulholland and Elwood (1982), Tranvik et al. (2009), Mendonça et al. (2017), and Anderson et al. (2020), respectively, earlier studies that are highly skewed towards cold high- and mid-latitude environments. These findings confirm recent studies that have supported the idea that there are important watershed controls on OC accumulation in lakes globally^10^. This is particularly true for the role of nutrient enrichment^8,9,11^ related to human-altered environments^7,12–16^ in regulating C storage. Growing evidence suggests higher lake OC burial with increasing primary production^17–19^ fuelled by both anthropogenic eutrophication^16,20–23^ and organic matter inputs from deforestation^24,25^.

Accordingly, our decadal accumulation rates derived from ^210^Pb dating in boreal lake sediments for last ~50-100 years indicated C accumulation ~7 times higher than those previously reported from radiocarbon analysis for the Holocene (last 10,000 years) at the same biome^12,26^. A similar order of magnitude difference in OC burial rates was found in sediment cores where these dating methods were used, *e.g.* ~15 times more in the Amazon floodplain lake Acarabixi^27^ and ~2 to 10 times more in the central margin of the Arabian Sea^28^ using ^210^Pb than radiocarbon. Despite the potential role of active diagenesis in surface sediments to overestimate lake C accumulation^29^, highly divergent OC burial rates have been attributed to the anthropogenic increases over the past decades^28,30^ and the lower uncertainty of ^210^Pb than radiocarbon dating on a time scale of up to a century^31,32^. Indeed, the relationships of non-flooded forest cover and OC burial rates in sediment profiles comparing above and below 10-cm layers showed the same results (Supp Figure 9), indicating no bias towards incomplete diagenesis in our conclusion.

However, our lower estimates of OC burial in human-altered versus conserved lakes also complemented earlier predictions derived from datasets that underrepresented low-latitude ecosystems (e.g., Stallard, 1998; Heathcote et al., 2015). We show here that tropical forest lakes are underrepresented in global data sets and are further shown here to bury more C per unit area annually, and sequester around ~23 and 8% more C than the eutrophic and hypereutrophic lakes previously reported^20,23^. The significant decrease in OC burial rates observed here in Amazonian lakes surrounded by lower forest cover would indicate an additional effect, related to initial peaks of C storage in lacustrine sediments after deforestation^21,34^ that they may be a transient feature that is followed by long-term C storage decline^35–38^. This dynamic is supported by the finding of loss of organic matter in response to reduced forest cover^39^ and subsequent increased soil exposure to biological remineralization^40^, also confirming recent and still scarce evidence on the mechanistic link between deforestation and changes in C accumulation in Amazon lakes^36^.

Indeed, the absence of tropical lakes surrounded by the most productive world biome on Earth (i.e., tropical humid forest) could have led to underestimates of the role of forest conservation in supporting C accumulation in aquatic sediments in previous reviews. The estimates of annual OC burial rates in global lakes here were comparable to ~2.5 and 3.0% of the terrestrial and ocean CO_2_ sink, respectively, or ~5% of the emissions from land-use change for the last decade^41^. Derived from a broad biome-weighted analysis, these results also indicated the critical role of forest cover to support OC burial in lacustrine ecosystems at the global scale. In particular, tropical lakes show a disproportionate C accumulation with respect to their smaller global land area, determined by the balance of more intense ecosystem processes at warm low latitudes that should be considered in global models, such as the overall C sink^2^ and the uptake of anthropogenic C^42^ from the atmosphere by terrestrial primary production, the OM remineralization in aquatic sediments^43^, or even the metabolic responses to recent warming^44^. Therefore, our novel findings reveal that global change science still needs to unravel a neglected loss of C burial capacity connecting broad terrestrial and aquatic environments, with potentially important implications of productive and threatened humid tropical forests to climate change mitigation.

**REFERENCES**

1. Messager, M. L., Lehner, B., Grill, G., Nedeva, I. & Schmitt, O. Estimating the volume and age of water stored in global lakes using a geo-statistical approach. *Nat. Commun.* **7**, 13603 (2016).

2. Pan, Y. *et al.* A large and persistent carbon sink in the world’s forests. *Science* **333**, 988–993 (2011).

3. Grace, J. & Malhi, Y. Global change: carbon dioxide goes with the flow. *Nature* vol. 416 594 (2002).

4. Malhi, Y. & Grace, J. Tropical forests and atmospheric carbon dioxide. *Trends Res. Ecol. Environ.* **15**, 332–337 (2000).

5. Mitra, S., Wassmann, R. & Vlek, P. L. G. An appraisal of global wetland area and its organic carbon stock. *Curr. Sci.* **88**, 25–35 (2005).

6. Olson, D. M. *et al.* Terrestrial Ecoregions of the World: A New Map of Life on Earth. *Bioscience* **51**, 933 (2001).

7. Mulholland, P. J. & Elwood, J. W. The role of lake and reservoir sediments as sinks in the perturbed global carbon cycle. *Tellus* **34**, 490–499 (1982).

8. Tranvik, L. J. *et al.* Lakes and reservoirs as regulators of carbon cycling and climate. *Limnol. Oceanogr.* **54**, 2298–2314 (2009).

9. Mendonça, R. *et al.* Organic carbon burial in global lakes and reservoirs. *Nat. Commun.* **8**, 1694 (2017).

10. Deevey Jr., E. S. Biogeochemistry of lakes: major substances. *Am. Soc. Limnol. Ocean. Spec. Symp.* **1**, 14–20 (1972).

11. Kortelainen, P. Content of Total Organic Carbon in Finnish Lakes and Its Relationship to Catchment Characteristics. *Can. J. Fish. Aquat. Sci.* **50**, 1477–1483 (1993).

12. Kortelainen, P. *et al.* Controls on the export of C, N, P and Fe from undisturbed boreal catchments, Finland. *Aquat. Sci.* **68**, 453–468 (2006).

13. Kastowski, M., Hinderer, M. & Vecsei, A. Long-term carbon burial in European lakes: Analysis and estimate. *Global Biogeochem. Cycles* **25**, n/a-n/a (2011).

14. Ferland, M. E., Del Giorgio, P. A., Teodoru, C. R. & Prairie, Y. T. Long-term C accumulation and total C stocks in boreal lakes in northern Québec. *Global Biogeochem. Cycles* **26**, (2012).

15. Anderson, N. J., D’andrea, W. & Frintz, S. C. Holocene carbon burial by lakes in SW Greenland. *Glob. Chang. Biol.* **15**, 2590–2598 (2009).

16. Anderson, N. J., Heathcote, A. J. & Engstrom, D. R. Anthropogenic alteration of nutrient supply increases the global freshwater carbon sink. *Sci. Adv.* **6**, eaaw2145 (2020).

17. Ahl, T. Effects of man-induced and natural loading of phosphorus and nitrogen on the large Swedish lakes. *SIL Proceedings, 1922-2010* **19**, 1125–1132 (1975).

18. Pennington, W. & Tutin, T. G. Responses of some British lakes to past changes in land use on their catchments. *SIL Proceedings, 1922-2010* **20**, 636–641 (1978).

19. Darchambeau, F., Sarmento, H. & Descy, J.-P. Primary production in a tropical large lake: The role of phytoplankton composition. *Sci. Total Environ.* **473**–**474**, 178–188 (2014).

20. Anderson, N. J., Bennion, H. & Lotter, A. F. Lake eutrophication and its implications for organic carbon sequestration in Europe. *Glob. Chang. Biol.* **20**, 2741–2751 (2014).

21. Anderson, N. J., Dietz, R. D. & Engstrom, D. R. Land-use change, not climate, controls organic carbon burial in lakes. *Proc. Biol. Sci.* **280**, 20131278 (2016).

22. Heathcote, A. J., Anderson, N. J., Prairie, Y. T., Engstrom, D. R. & del Giorgio, P. A. Large increases in carbon burial in northern lakes during the Anthropocene. *Nat. Commun.* **6**, 10016 (2015).

23. Sobek, S. *et al.* Organic carbon burial efficiency in lake sediments controlled by oxygen exposure time and sediment source. *Limnol. Oceanogr.* **54**, 2243–2254 (2009).

24. Bormann, F. H., Likens, G. E., Siccama, T. G., Pierce, R. S. & Eaton, J. S. The Export of Nutrients and Recovery of Stable Conditions Following Deforestation at Hubbard Brook. *Ecol. Monogr.* **44**, 255–277 (1974).

25. Kortelainen, P. *et al.* Carbon evasion/accumulation ratio in boreal lakes is linked to nitrogen. *Global Biogeochem. Cycles* **27**, 363–374 (2013).

26. Kortelainen, P., Pajunen, H., Rantakari, M. & Saarnisto, M. A large carbon pool and small sink in boreal Holocene lake sediments. *Glob. Chang. Biol.* **10**, 1648–1653 (2004).

27. Moreira, L. S., Moreira-Turcq, P. F., Cordeiro, R. C. & Turcq, B. J. Reconstituição paleoambiental do Lago Santa Ninha, Várzea do Lago Grande de Curuai, Pará, Brasil. *Acta Amaz.* **39**, 609–616 (2009).

28. Somayajulu, B. L. K., Bhushan, R., Sarkar, A., Burr, G. S. & Jull, A. J. T. Sediment deposition rates on the continental margins of the eastern Arabian Sea using 210Pb, 137Cs and 14C. *Sci. Total Environ.* **237**–**238**, 429–439 (1999).

29. Hanson, P. C., Pace, M. L., Carpenter, S. R., Cole, J. J. & Stanley, E. H. Integrating Landscape Carbon Cycling: Research Needs for Resolving Organic Carbon Budgets of Lakes. *Ecosystems* **18**, 363–375 (2015).

30. Cordeiro, R. C. *et al.* Environmental and anthropic variabilities at Guanabara Bay (Brazil): A comparative perspective of metal depositions in different time scales during the last 5,500 yrs. *Chemosphere* **267**, 128895 (2021).

31. Appleby, P. G. Dating recent sediments by 210 pb: problems and solutions. *Stuk a-145* 7–24 (1998).

32. Godwin, H. Half-life of Radiocarbon. *Nature* **195**, 984–984 (1962).

33. Stallard, R. F. Terrestrial sedimentation and the carbon cycle: Coupling weathering and erosion to carbon burial. *Global Biogeochem. Cycles* **12**, 231–257 (1998).

34. Li, X., Yuan, H., Li, N. & Song, J. Organic carbon source and burial during the past one hundred years in Jiaozhou Bay, North China. *J. Environ. Sci.* **20**, 551–557 (2008).

35. Schelske, C. L., Peplow, A., Brenner, M. & Spencer, C. N. Low-background gamma counting: applications for210Pb dating of sediments. *J. Paleolimnol.* **10**, 115–128 (1994).

36. Sanders, L. M. *et al.* Historic carbon burial spike in an Amazon floodplain lake linked to riparian deforestation near Santarém, Brazil. *Biogeosciences* **15**, 447–455 (2018).

37. Leithold, E. L., Perkey, D. W., Blair, N. E. & Creamer, T. N. Sedimentation and carbon burial on the northern California continental shelf: The signatures of land-use change. *Cont. Shelf Res.* **25**, 349–371 (2005).

38. Ruiz-Fernández, A. C., Hillaire-Marcel, C., Páez-Osuna, F., Ghaleb, B. & Caballero-Miranda, M. 210Pb chronology and trace metal geochemistry at Los Tuxtlas, Mexico, as evidenced by a sedimentary record from the Lago Verde crater lake. *Chinese J. Geochemistry* **25**, 17–17 (2006).

39. Durigan, M. R. *et al.* Soil organic matter responses to anthropogenic forest disturbance and land use change in the eastern Brazilian Amazon. *Sustain.* **9**, (2017).

40. Kukla, J. *et al.* The effect of traditional slash-and-burn agriculture on soil organic matter, nutrient content, and microbiota in tropical ecosystems of Papua New Guinea. *L. Degrad. Dev.* **30**, 166–177 (2019).

41. Le Quéré, C. *et al.* Global Carbon Budget 2018. *Earth Syst. Sci. Data* **10**, 2141–2194 (2018).

42. Fernández-Martínez, M. *et al.* Global trends in carbon sinks and their relationships with CO2 and temperature. *Nat. Clim. Chang.* **9**, 73–79 (2019).

43. Marotta, H., Pinho, L. & Gudasz, C. Greenhouse gas production in low-latitude lake sediments responds strongly to warming. *Nat. Clim. Chang.* **4**, 11–14 (2014).

44. Dillon, M. E., Wang, G. & Huey, R. B. Global metabolic impacts of recent climate warming. *Nature* **467**, 704–706 (2010).
